# Independent factors affecting hemorrhagic and ischemic stroke in patients aged 40–69 years: a cross-sectional study

**DOI:** 10.1186/s12872-022-02625-6

**Published:** 2022-04-21

**Authors:** Takahisa Mori, Kazuhiro Yoshioka, Yuhei Tanno, Shigen Kasakura

**Affiliations:** Department of Stroke Treatment, Shonan Kamakura General Hospital, Okamoto 1370-1, Kamakura City, Kanagawa 247-8533 Japan

**Keywords:** Acute ischemic stroke, Blood pressure, Eicosapentaenoic acid, Intracerebral hemorrhage

## Abstract

**Background:**

Intracerebral hemorrhage (ICH) and acute ischemic stroke (AIS) have common vascular risk factors; however, ICH often occurs in adults aged < 70 years. Intracerebral hemorrhage and AIS in adults aged < 70 years should be preventable; however, it is unclear why different subtypes of ICH or AIS occur among adults aged < 70 years with vascular risk factors. This study aimed to identify independent variables for ICH or AIS onset in patients aged < 70 years.

**Methods:**

We included patients aged 40–69 years who experienced ICH or AIS between August 2016 and July 2019. Patients aged < 40 years were excluded because other diseases, rather than vascular risk factors, are often associated with stroke etiology in this age group. Data on age, systolic blood pressure (SBP), serum lipids, and serum fatty acid levels were compared between patients with ICH and those with AIS. In addition, we conducted multivariable logistic regression analyses to identify independent factors among the variables, such as blood pressure or biomarkers, with significant differences between the two groups.

**Results:**

Of the 1252 acute stroke patients screened, 74 patients with ICH and 149 patients with AIS met the inclusion criteria. After excluding variables with multicollinearity, SBP, glycated hemoglobin (HbA1c), and eicosapentaenoic acid (EPA) proportion (%) of total fatty acids were identified as independent factors affecting ICH and AIS. The SBP and EPA% threshold values for ICH compared to AIS were ≥ 158 mmHg and ≤ 2.3%, respectively. The HbA1c threshold value for AIS compared to ICH was ≥ 6.1%.

**Conclusions:**

Systolic blood pressure, HbA1c, and EPA%, were independent factors between ICH and AIS. Patients aged 40–69 years with high SBP and low EPA% were at a higher risk of ICH than AIS, and those with a high HbA1c were at a higher risk of AIS than ICH.

**Supplementary Information:**

The online version contains supplementary material available at 10.1186/s12872-022-02625-6.

## Background

Intracerebral hemorrhage (ICH) and acute ischemic stroke (AIS) have common vascular risk factors such as hypertension, dyslipidemia, diabetes mellitus, and obesity [1–3]; however, the reason for the onset of different subtypes, ICH or AIS, is unclear. The mean age for ICH and AIS onset in Japan has been identified as the 60 s and 70 s, respectively [4, 5]. Moreover, the mortality rates within 28 days for ICH and AIS were 18.9% and 6.9%, respectively, and the rates of having modified Rankin scale scores of 4–6 (severe disability or death) at discharge were 63.0% and 40.5% in first-ever ICH and AIS, respectively [6, 7]. Intracerebral hemorrhage often occurs in adults aged < 70 years, and patients with ICH experience more disabilities than those with AIS. Therefore, ICH and AIS in adults aged < 70 years should be preventable; however, it is unclear why different subtypes of ICH or AIS occur among adults aged < 70 years with vascular risk factors.

The etiology of stroke patients aged < 40 years frequently consists of vascular malformation, moyamoya disease, or autoimmune disease [8, 9]. Therefore, patients aged < 40 years should be excluded when identifying independent factors for ICH or AIS among adults aged < 70 years who are susceptible to vascular risk factors [10].

Elevated blood pressure has been identified as the greatest risk factor for hemorrhagic or ischemic stroke [11]. Additionally, diabetes is an independent risk factor for ischemic stroke [12], and the serum cholesterol level is inversely related to the risk of death from hemorrhagic stroke and positively related to death from ischemic stroke and total cardiovascular disease [13]. Serum levels and proportion (%) of fatty acid (FA) are associated with AIS or ICH [14–18]. Triglycerides (TG) comprise glycerol and three fatty acids (FAs); FAs affect the quality of TG, and dietary TG affects serum FA levels [14]. A high level of n-3 polyunsaturated fatty acids (n-3 PUFAs) reduces the risk of ischemic stroke [15]. High levels of saturated FA, n-9 monounsaturated FA, and n-6 PUFA increase the risk of ischemic stroke [16]. A high level of dihomo-gamma-linolenic acid (DGLA) is associated with early-onset ICH or AIS [17, 18]. The serum percentage of docosahexaenoic acid (DHA) is associated with early-onset AIS [18], and that of eicosapentaenoic acid (EPA) is associated with early-onset ICH [17]. Therefore, DGLA, EPA%, and DHA% are significant risk factors for early-onset ICH or AIS [17, 18].

Serum FA levels at arrival performed under non-fasting conditions are associated with the dietary intake of TG immediately before stroke onset. Therefore, this retrospective cross-sectional study aimed to investigate the reason for onset of different stroke subtypes among adults aged 40–69 years with vascular risk factors and identify independent factors for ICH or AIS onset.

## Methods

### Patients

We included patients admitted to our institution within 24 h of stroke onset between August 2016 and July 2019. We excluded the following patients: (1) aged < 40 years or ≥ 70 years, (2) those who did not undergo an examination of serum DGLA level, EPA%, and DHA% at arrival, (3) those with a severe disability or underweight status at admission, or (4) those with the etiology of vascular malformation, moyamoya disease, autoimmune disease, or cancer-related thrombosis. We defined a severe disability as a pre-hospital modified Rankin scale score ≥ 3 [6] and underweight status as a body mass index < 18.5 (kg/m^2^), because patients with these conditions suffer from possible malnutrition. Investigation of DGLA, EPA%, and DHA% were necessary to investigate the reason for the different onset of stroke subtypes, namely, early-onset ICH or AIS [17, 18].

### Data collection

We collected patient data on anthropometric variables, blood pressure, National Institutes of Health Stroke Scale (NIHSS) score [19], history of medications for hypertension, diabetes, or dyslipidemia, and biomarkers relevant to vascular risk factors such as glucose, glycated hemoglobin (HbA1c), serum concentration of DGLA, and proportions (%) of EPA and DHA, total cholesterol (TC), high-density lipoprotein cholesterol (HDL), low-density lipoprotein cholesterol (LDL), and TG, at admission. As reported previously, serum TG, HDL, and FA levels were also measured [18].

### Statistical analysis

The chi-square test was used to compare the categorical variables between the ICH and AIS groups. Non-normally distributed continuous variables were expressed as medians and interquartile ranges and the Wilcoxon rank-sum test was used to compare continuous variables between the ICH and AIS groups through normal approximation. A multivariable comparison test was used to compare all possible pairs of variables with significant differences between the two groups. Spearman’s rank correlation coefficient (*r*_*s*_) was used to measure the strength of the relationships between non-normally distributed variables with significant differences between the two groups. We truncated the *r*_*s*_ below the second decimal point and defined: 0 ≤|*r*_*s*_|< 0.1 as no correlation, 0.1 ≤|*r*_*s*_|< 0.4 as a weak correlation, 0.4 ≤|*r*_*s*_|< 0.6 as a moderate correlation, and 0.6 ≤*|r*_*s*_| as a strong correlation. Variables with significant differences in the chi-square test or the Wilcoxon rank-sum test between the two groups were candidates for multivariable logistic regression analyses. We adopted the variable candidate with a smaller or the smallest probability (*p*) value or a larger or the largest absolute value of the Wilcoxon rank-sum test statistic (|z|) between or among variable candidates with mutually strong correlations (|*r*_*s*_|≥ 0.6). We defined multicollinearity as variance inflation factor ≥ 3, indicating that the multiple correlation coefficient is > 0.8. We conducted multivariable logistic regression analyses using variables without multicollinearity. We estimated the threshold values of independent ICH-onset variables using the receiver operating characteristic curves. Furthermore, we analyzed the data stratified according to age (40–54 vs. 55–69 years) as secondary analysis. We reported *p*-values up to four decimal places (< 0.0001). We presented *p* values using two-sided tests. Significance was set at *p* value < 0.05. JMP software (version 16.2; SAS Institute, Cary, NC, USA) was used for all statistical analyses.

## Results

A total of 1152 acute stroke patients were admitted to our stroke center during the study period; of these patients, 74 with ICH (33.2%) and 149 with AIS (66.8%) met our inclusion criteria (Additional file 1). Patient characteristics at the time of admission are summarized in Table 1. There were no patients with stroke caused by vascular malformation, moyamoya disease, autoimmune disease, or cancer-related thrombosis who met the inclusion criteria. The etiology of 74 patients with ICH was hypertension (Additional file 2), although only 19 (25.7%) of 74 patients were treated with antihypertensive drugs (Table 1). Aside from medication history and NIHSS scores: four variables showed significant differences between the two groups. Patients in the ICH group had higher SBP and DBP and a lower EPA% level than those in the AIS group. In addition, patients in the AIS group had a higher HbA1c level than those in the ICH group.

**Table 1:** Patient characteristics

	ICH	AIS	|z| or chi	*p*-value
n	74	149		
Age (years)	58.5 (51–65)	62 (54.5–67)	1.80	0.0710
Male sex	51 (68.9%)	101 (67.8%)	0.03	0.8640
Height (cm)	166 (159.8–170)	165 (160–171)	0.01	0.9886
Bodyweight (kg)	61.1 (59.5–78.5)	66 (58–76.1)	0.31	0.7600
BMI (kg/m^2^)	24.2 (21.8–26.9)	24.5 (21.8–26.8)	0.15	0.8843
SBP (mmHg)	183.5 (162–209)	157 (138–181)	5.23	**< 0.0001**
DBP (mmHg)	111.5 (97.8–129)	96 (82.5–111.5)	5.09	**< 0.0001**
Glucose (mmol/L)	6.8 (5.7–8.4)	6.5 (5.8–8.3)	0.03	0.9789
HbA1c (%) (NGSP)	5.7 (5.5–6.15)	5.9 (5.6–6.6)	2.60	**0.0094**
TC (mmol/L)	5.48 (4.93–6.14)	5.33 (4.73–6.09)	0.66	0.5063
HDL (mmol/L)	1.55 (1.22–1.94)	1.42 (1.18–1.76)	1.88	0.0596
TG (mmol/L)	1.56 (0.86–2.45)	1.25 (0.84–2.17)	0.82	0.4118
LDL (mmol/L)	3.09 (2.23–3.57)	3.12 (2.47–3.62)	0.60	0.5466
DGLA (μmol/L)	116.9 (97.2–146.8)	108.6 (80.7–138.9)	1.72	0.0862
EPA%	1.45(0.9–2.3)	1.80 (1.1–2.7)	1.99	**0.0469**
DHA%	3.8 (2.8–5.03)	4.0 (3.1–5)	0.82	0.4133
History of statin use	9 (12.2%)	28 (18.8%)	1.64	0.2003
History of DL drugs	8 (10.8%)	32 (21.5%)	4.10	**0.0429**
History of anti-HT drugs	19 (25.7%)	52 (34.9%)	1.98	0.1595
History of diabetes drugs	3 (4.1%)	19 (12.8%)	4.83	**0.0280**
NIHSS at admission	9 (3–18)	2 (1–5)	6.03	**< 0.0001**
NIHSS at discharge	5 (1.5–12)	1 (0–3)	5.55	**< 0.0001**
Hospitalization, days	8 (8–9)	8 (7–8)	1.44	0.1497
In-hospital death, N	2 (2.70%)	1 (0.67%)	1.42	0.2333

All values except for categorical data are represented as median (interquartile range). Boldface indicates statistical significance (*p* < 0.05)

*AIS* acute ischemic stroke, *BMI* body mass index, *chi* chi-square value, *DL* dyslipidemia, *EPA* eicosapentaenoic acid, *DHA* docosahexaenoic acid, *DBP* diastolic blood pressure, *DGLA* dihomo-gamma-linolenic acid, *HbA1c* glycated hemoglobin, *HDL* high-density lipoprotein cholesterol, *HT* hypertensive, *ICH* intracerebral hemorrhage, *LDL* low-density lipoprotein cholesterol, *NGSP* National Glycohemoglobin Standardization Program, *n* number, *NIHSS* National Institutes of Health Stroke Scale score, *p* probability, *SBP* systolic blood pressure, *TC* total cholesterol, *TG* triglycerides, *|z|* absolute value of the Wilcoxon rank-sum test statistic

No differences were found in age; sex; anthropometric variables; concentrations of glucose, TC, LDL, TG, HDL, and DGLA; or DHA% between the two groups. The history of diabetes or dyslipidemia drugs was higher in the AIS group than in the ICH group. The median NIHSS scores at admission and discharge were higher in the ICH group than in the AIS group, and patients with ICH had more disabilities at discharge than patients with AIS. Among the four variables with significant differences between the two groups that were assessed, a strong positive correlation was found between SBP and DBP (Additional file 3). We adopted SBP as the variable candidate for multivariable logistic regression because the |z| of SBP was larger than that of DBP (Table 1). After confirming that the variance inflation factors of three variable candidates were < 3 (Additional file 4), multivariable logistic regression analysis showed that high SBP and low EPA% were independent factors of ICH, rather than AIS, and that a high HbA1c was an independent factor for AIS, rather than ICH (Table 2).

**Table 2:** Multivariable logistic regression for intracerebral hemorrhage onset compared with acute ischemic stroke onset

	Odds ratio	*p* value	AUC	BIC
		**< 0.0001**	0.752	261
SBP	1.02 (1.01–1.03)	**< 0.0001**		
HbA1c	0.60 (0.41–0.82)	**0.0009**		
EPA%	0.80 (0.62–0.99)	**0.0491**		

Boldface indicates statistical significance (*p* < 0.05)

*AUC* area under the curve, *BIC* Bayesian information criterion, *EPA* eicosapentaenoic acid, *HbA1c* glycated hemoglobin, *p* probability, *SBP* systolic blood pressure

The receiver operating characteristic curves of ICH from AIS demonstrated that the threshold values of SBP and EPA% were ≥ 158 mmHg, and ≤ 2.3%, respectively, and the threshold value of HbA1c for AIS from ICH was ≥ 6.1% (Tables 3, 4).

**Table 3:** Threshold values for ICH compared to AIS

	Sens (%)	Spec (%)	Odds ratio	*p* value	AUC	BIC
SBP (≥ 158 vs. < 158) mmHg	85.1	53.7	1.02 (1.01–1.03)	< 0.0001	0.715	268
EPA% (≤ 2.3 vs. > 2.3)	78.4	36.2	0.77 (0.61–0.95)	0.0121	0.582	288

*AUC* area under the curve, *BIC* Bayesian information criterion, *EPA* eicosapentaenoic acid, *p* probability, *SBP* systolic blood pressure, *Sens* sensitivity, *Spec* specificity, *%* weight percentage of total fatty acids

**Table 4:** Threshold value for AIS compared with ICH

	Sens (%)	Spec (%)	Odds ratio	*p* value	AUC	BIC
HbA1c (≥ 6.1 vs. < 6.1)%	72.6	44.3	0.65 (0.45–0.89)	0.0047	0.607	284

*AUC* area under the curve, *BIC* Bayesian information criterion, *HbA1c* glycated hemoglobin, *p* probability, *Sens* sensitivity, *Spec* specificity, *%* weight percentage of total fatty acids

As a result of secondary analysis, for patients aged 40–54 years, the ratio of ICH was 0.412 (26/63), and blood pressure, high SBP or DBP, was the only factor for ICH. In the subgroup analysis patients aged 55–69 years, the ratio of ICH decreased to 0.3 (48/160), high SBP and DBP were factors for ICH, and high HbA1c and low HDL levels were significant factors for AIS. As the age advanced, the ratio of ICH decreased, and diabetes or dyslipidemia became significant for AIS onset. In the subgroup analysis for age, only six (23.1%) of 26 patients aged 40–54 years and 13 (27.1%) of 48 patients aged 55–69 years with ICH took antihypertensive drugs pre-ICH (ns). Additionally, only four (10.8%) of 37 patients aged 40–54 years and 48 (42.9%) of 112 patients aged 55–69 years with AIS took antihypertensive drugs pre-AIS (*p* = 0.0001). In the subgroup analysis for age, only two (5.4%) of 37 patients aged 40–54 years and 30 (26.8%) of 112 patients aged 55–69 years with AIS took DL drugs pre-AIS (*p* = 0.0023). (Additional files 5 and 6).

## Discussion

Our results demonstrated that independent factors for ICH, but not AIS, were high SBP and low EPA%. Additionally, an independent factor for AIS, but not ICH, was high HbA1c. Thus, patients aged 40–69 years with high blood pressure and low EPA% were at a higher risk of ICH than AIS, and patients aged 40–69 years with high HbA1c were at a higher risk of AIS than ICH. Among 74 hypertensive patients with ICH, only 19 patients (25.7%) took antihypertensive drugs before experiencing ICH, suggesting a lack of appropriate blood pressure control. The threshold value of EPA% for ICH compared to AIS was ≤ 2.3%, and the threshold value of HbA1c for AIS compared to ICH was ≥ 6.1%.

Low compliance of antihypertensive drugs may be associated with ICH onset for patients aged 40–54 years and 55–69 years. In patients aged 40–54 years, blood pressure was the only significant factor for ICH, and the ratio of ICH was higher than that in patients aged 55–69 years, suggesting the importance of blood pressure control to prevent ICH in patients aged 40–54 years. On the other hand, HbA1c, antihypertensive drug use, or DL drug use increased in patients with AIS with advancing age. The ratio of AIS was higher in patients aged 55–69 years than patients aged 40–54 years, suggesting the importance of blood pressure control, HbA1c management, or dyslipidemia treatment to prevent AIS. Diabetes is an independent risk of ischemic stroke [12], and serum cholesterol level is positively related to death from ischemic stroke and total cardiovascular disease [13].

The occurrence of hypertension is proportional to salt intake [20]. In addition, genetics is a risk factor for hypertension [21]. Instant noodles, which contain a high volume of salt and high concentration of refined carbohydrates, contribute to high diastolic blood pressure, high TG level, and high fasting blood glucose levels even among college students [22]. Patients with impaired glucose tolerance consuming a diet containing > 45% carbohydrates show a greater postprandial glucose spike [23]. The serum EPA level is positively correlated to fish intake [14, 24]. Therefore, a low EPA% suggests a low dietary intake of fish. A diet comprising low amounts of fish with a large salt intake may cause early-onset ICH compared to AIS. Patients aged 40–69 with AIS had a higher HbA1c level and a high frequency of dyslipidemia and intake of drugs to treat diabetes. Therefore, a large lipid and carbohydrate diet may cause early-onset AIS compared to ICH.

A reduction in sodium intake lowers blood pressure [25]. Compared with diuretics and β-blockers, calcium-channel blockers might protect more against stroke than against myocardial infarction [26]. According to recommendations for patients with diabetes [27], reducing carbohydrate intake has been the best medical nutrition for improving hyperglycemia. Dietary guidance focuses on consuming fish and low-fat or fat-free dairy products to prevent atherosclerotic cardiovascular disease [28]. Thus, n-3 PUFAs can reduce the incidence of stroke [28–33].

In this study, no difference was found in the DGLA level between ICH and AIS patients; however, a high DGLA level is significantly associated with ICH onset at a younger age [17]. Therefore, reducing salt or DGLA intake and consuming fish is necessary to prevent ICH in patients aged 40–69 years. In addition, following the traditional Japanese diet, which mainly comprises fish, soybeans, soy products, seaweed, mushroom, konjac, and unrefined cereals, with low amounts of animal fat, meat, poultry with fat, and sweets, including desserts and snacks, was effective in increasing serum n-3 PUFA% and decreasing serum n-6 PUFA% [34]. Furthermore, high consumption of isoflavones was associated with a reduced risk of ischemic stroke and myocardial infarction among postmenopausal women [35], and the traditional Japanese diet was effective in preventing the risk factors of coronary artery disease [36]. Therefore, the traditional Japanese diet may effectively prevent ICH or AIS. A lifestyle of daily intake of a high volume of salt and high concentration of refined carbohydrates requires rapid and significant improvement among young adults aged < 40 years to prevent stroke onset in the 40–60 s.

For prevention of ICH, blood pressure should be controlled in adults aged 40–69 years, particularly those aged 40–54 years, and dietary EPA% should be increased for adults aged 40–69 years to prevent ICH. In addition, for prevention of AIS, blood pressure should be controlled, HbA1c should be managed, or dyslipidemia should be treated in adults aged 40–69, particularly those aged 55–69 years.

### Limitations

This study has several limitations. First, a small number of patients were included, and the study was retrospective and cross-sectional. A cross-sectional study has reverse causality. Second, our study did not investigate dietary intake of sodium, FA, or carbohydrates. Third, because most of the patients were Japanese, generalization of the study outcomes to non-Japanese populations may not be possible. There might be racial differences in the association of ICH-related factors with the threshold values of factors for ICH. Fourth, data on basic demographic information (education, income, marriage), lifestyles (smoking, drinking, physical activity, diet), and family history of acute stroke were not collected, which may have led to a bias in the results. Fifth, our patients did not undergo analysis for the genome associated with early-onset high blood pressure or high HbA1c. A prospective randomized control study using a food frequency questionnaire, blood pressure measurement, and serum FA levels is needed to determine the effects of sodium and fish on ICH or AIS and prevention of stroke in patients aged 40–69 years.

## Conclusions

Systolic blood pressure, HbA1c, and EPA% were independent factors with significant differences between ICH and AIS in patients aged 40–69 years. Patients with high SBP and low EPA% were at a higher risk of ICH, and patients with high HbA1c were at a higher risk of AIS. Further studies are warranted to investigate the influence of race, ethnicity, or genome and the effects of changing lifestyle on the prevention of ICH and AIS in patients aged 40–69 years.

## Supplementary Information

**Additional file 1**. Flow chart of patient selection for the analysis

**Additional file 2**. The etiology of acute stroke

**Additional file 3**. Spearman’s rank correlation coefficients between individual variables with significant differences between the study groups

**Additional file 4**. Variance inflation factors of three variables for multivariate analysis

**Additional file 5**. Characteristics of patient aged 40-54 years

**Additional file 6**. Characteristics of patient aged 55-69 years

## Data Availability

The datasets generated and/or analyzed during the current study are available from the corresponding author on reasonable request.

## Abbreviations

DGLA: Dihomo-gamma-linolenic acid
DHA: Docosahexaenoic acid
EPA: Eicosapentaenoic acid
FA: Fatty acid
NIHSS: National Institutes of Health Stroke Scale
PUFA: Polyunsaturated fatty acids
SBP: Systolic blood pressure
