# Supplementary material for: Independent factors affecting hemorrhagic and ischemic stroke in patients aged 40–69 years: a cross-sectional study
Source: BMC Cardiovasc Disord. 2022 Apr 21;22:189. doi: 10.1186/s12872-022-02625-6 (PMC9027078; doi:10.1186/s12872-022-02625-6)
Supplement: Supplementary file 1 — Additional file 1. Flow chart of patient selection for the analysis [file 12872_2022_2625_MOESM1_ESM.docx]

**Additional files**

Independent factors affecting hemorrhagic and ischemic stroke in patients aged 40-69 years: a cross-sectional study

**
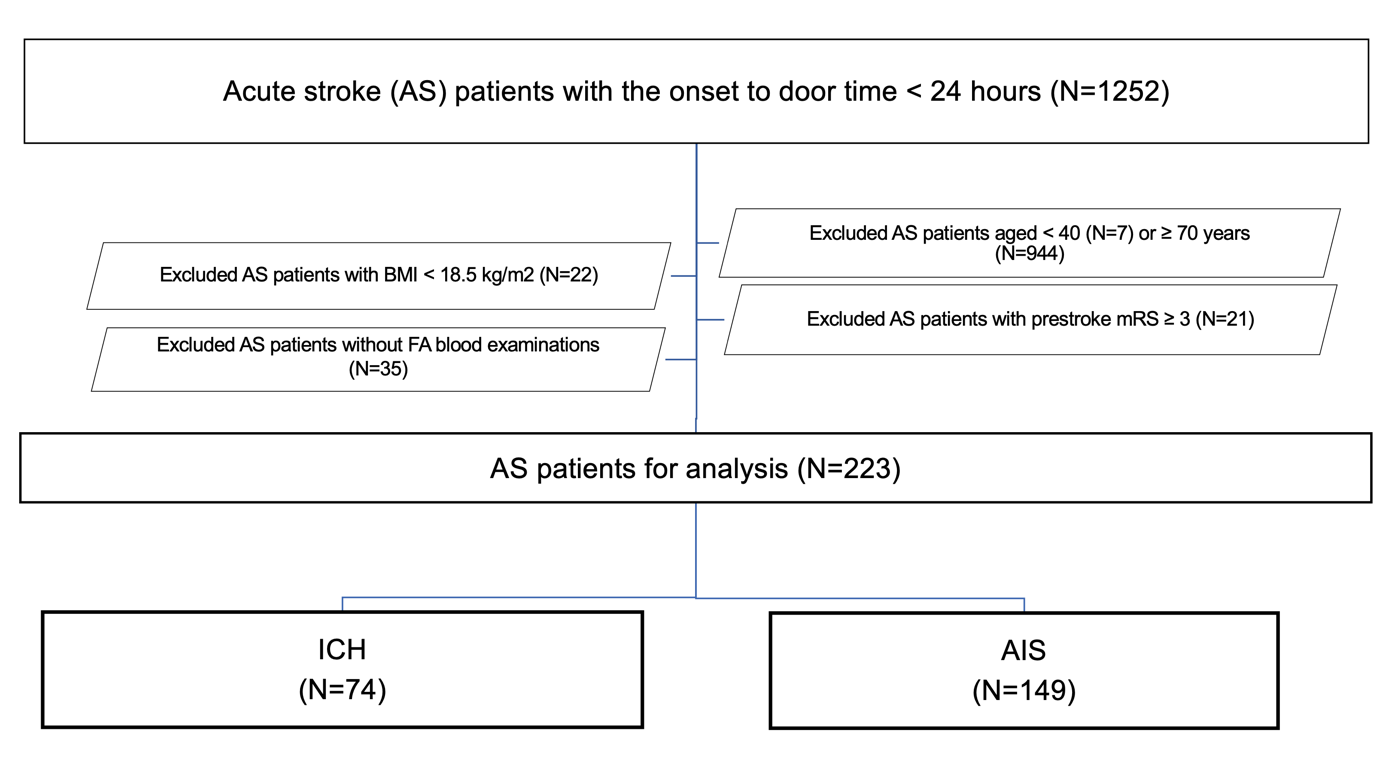
**

**Additional file 1. Flow chart of patient selection for the analysis**

AIS, acute ischemic stroke; BMI, body mass index; ICH, intracerebral hemorrhage; mRS, modified Rankin scale score; n, number.
