# Supplementary material for: Independent factors affecting hemorrhagic and ischemic stroke in patients aged 40–69 years: a cross-sectional study
Source: BMC Cardiovasc Disord. 2022 Apr 21;22:189. doi: 10.1186/s12872-022-02625-6 (PMC9027078; doi:10.1186/s12872-022-02625-6)
Supplement: Supplementary file 2 — Additional file 2. The etiology of acute stroke [file 12872_2022_2625_MOESM2_ESM.docx]

Additional file 2. The etiology of acute stroke

| etiology | n |
| --- | --- |
| cardioembolism | 15 |
| Large artery atherosclerosis | 87 |
| Small vessel occlusion | 44 |
| Transient ischemic attack | 3 |
| Hypertensive intracerebral hemorrhage | 74 |
