# Supplementary material for: Independent factors affecting hemorrhagic and ischemic stroke in patients aged 40–69 years: a cross-sectional study
Source: BMC Cardiovasc Disord. 2022 Apr 21;22:189. doi: 10.1186/s12872-022-02625-6 (PMC9027078; doi:10.1186/s12872-022-02625-6)
Supplement: Supplementary file 3 — Additional file 3. Spearman’s rank correlation coefficients between individual variables with significant differences between the study groups [file 12872_2022_2625_MOESM3_ESM.docx]

Additional file 3. Spearman’s rank correlation coefficients between individual variables with significant differences between the study groups

| *r*_s_ | SBP | DBP | A1c | EPA% |
| --- | --- | --- | --- | --- |
| SBP |  | **0.70** | 0.03 | -0.10 |
| DBP | **0.70** |  | 0.00 | -0.15 |
| A1c | 0.03 | 0.00 |  | 0.10 |
| EPA% | -0.10 | -0.15 | 0.10 |  |

Boldface indicates moderate or strong correlation. A1c, glycated hemoglobin; DBP, diastolic blood pressure; EPA, eicosapentaenoic acid; *r*_s、_Spearman’s rank correlation coefficient; SBP, systolic blood pressure.
