# Supplementary material for: Independent factors affecting hemorrhagic and ischemic stroke in patients aged 40–69 years: a cross-sectional study
Source: BMC Cardiovasc Disord. 2022 Apr 21;22:189. doi: 10.1186/s12872-022-02625-6 (PMC9027078; doi:10.1186/s12872-022-02625-6)
Supplement: Supplementary file 4 — Additional file 4. Variance inflation factors of three variables for multivariate analysis [file 12872_2022_2625_MOESM4_ESM.docx]

**Additional file 4.** Variance inflation factors of three variables for multivariate analysis

|  | VIF |
| --- | --- |
| SBP | 1.0 |
| A1c | 1.0 |
| EPA% | 1.0 |

A1c, glycated hemoglobin; EPA, eicosapentaenoic acid; SBP, systolic blood pressure; VIF, variance inflation factor
