# Supplementary material for: Independent factors affecting hemorrhagic and ischemic stroke in patients aged 40–69 years: a cross-sectional study
Source: BMC Cardiovasc Disord. 2022 Apr 21;22:189. doi: 10.1186/s12872-022-02625-6 (PMC9027078; doi:10.1186/s12872-022-02625-6)
Supplement: Supplementary file 5 — Additional file 5. Characteristics of patient aged 40-54 years [file 12872_2022_2625_MOESM5_ESM.docx]

**Additional file 5.** Characteristics of patient aged 40-54 years

|  | ICH | AIS | \|z\| or chi | *p*-value |
| --- | --- | --- | --- | --- |
| n | 26 | 37 |  |  |
| Age (years) | 49 (41–54) | 49 (40–54) | 0.13 | 0.8941 |
| Male sex | 21 (80.8%) | 23 (62.2%) | 2.60 | 0.1069 |
| BMI (kg/m2) | 24.8 (21.8–29.5) | 25.1 (21.5–28.8) | 0.09 | 0.9277 |
| SBP (mmHg) | 175 (162–211) | 156 (137–173) | 3.36 | **0.0008** |
| DBP (mmHg) | 115 (96–136) | 94 (80–112) | 3.28 | **0.0010** |
| Glucose (mmol/L) | 6.6 (5.7–7.6) | 6.7 (5.8–8.0) | 0.52 | 0.6054 |
| A1c (%)(NGSP) | 5.6 (5.4–6.0) | 5.7 (5.5–6.3) | 1.1 | 0.2798 |
| TC (mmol/L) | 5.26 (4.83-5.86) | 5.15 (4.62-5.82) | 0.53 | 0.5957 |
| HDL (mmol/L) | 1.37 (1.16–1.76) | 1.33 (1.10–1.55) | 0.84 | 0.4022 |
| TG (mmol/L) | 1.92 (0.78–3.31) | 1.48 (0.83–2.88) | 0.42 | 0.6772 |
| LDL (mmol/L) | 2.91 (2.08–3.47) | 2.69 (2.38–3.49) | 0.05 | 0.9610 |
| DGLA (μmol/L) | 139.2 (101.0–177.9) | 114.8 (80.4–148.2) | 1.62 | 0.1053 |
| EPA% | 1.0(0.8–2.0) | 1.4 (0.8–1.7) | 0.29 | 0.7690 |
| DHA% | 3.2 (2.6–4.5) | 3.5 (2.4–4.3) | 0.31 | 0.7584 |
| History of DL drugs | 2 (7.7%) | 2 (5.4%) | 0.13 | 0.7160 |
| History of anti-HT drugs | 6 (23.1%) | 4 (10.8%) | 1.69 | 0.1931 |
| History of diabetes drugs | 0 (0%) | 3 (8.1%) | 3.30 | 0.0694 |
| NIHSS at admission | 11.5 (4.3–17.8) | 3 (1–5) | 3.36 | **0.0008** |
| NIHSS at discharge | 7 (1.3–12) | 1 (0–3) | 3.39 | **0.0007** |

All values except for categorical data are represented as median (interquartile range). Boldface indicates statistical significance (*p*<0.05). A1c, glycated hemoglobin; AIS, acute ischemic stroke; BMI, body mass index; chi, chi-square value; DL, dyslipidemia; EPA, eicosapentaenoic acid; DHA, docosahexaenoic acid; DBP, diastolic blood pressure; DGLA, dihomo-gamma-linolenic acid; HDL, high-density lipoprotein cholesterol; HT, hypertensive; ICH, intracerebral hemorrhage; LDL, low-density lipoprotein cholesterol; NGSP, National Glycohemoglobin Standardization Program; n, number; NIHSS, National Institutes of Health Stroke Scale score; *p*, probability; SBP, systolic blood pressure; TC, total cholesterol; TG, triglycerides; |z|, absolute value of the Wilcoxon rank-sum test statistic.
