# Supplementary material for: Independent factors affecting hemorrhagic and ischemic stroke in patients aged 40–69 years: a cross-sectional study
Source: BMC Cardiovasc Disord. 2022 Apr 21;22:189. doi: 10.1186/s12872-022-02625-6 (PMC9027078; doi:10.1186/s12872-022-02625-6)
Supplement: Supplementary file 6 — Additional file 6. Characteristics of patient aged 55-69 years [file 12872_2022_2625_MOESM6_ESM.docx]

**Additional file 6.** Characteristics of patient aged 55-69 years

|  | ICH | AIS | \|z\| or chi | *p*-value |
| --- | --- | --- | --- | --- |
| n | 48 | 112 |  |  |
| Age (years) | 64 (60–67) | 65 (61–68) | 0.97 | 0.3321 |
| Male sex | 30 (62.5%) | 78 (69.6%) | 0.771 | 0.3798 |
| BMI (kg/m2) | 23.8 (21.7–26.1) | 24.2 (21.8–26.7) | 0.22 | 0.8261 |
| SBP (mmHg) | 188 (163–207) | 157 (138–183) | 4.09 | **< 0.0001** |
| DBP (mmHg) | 107 (98–129) | 97 (83.3–111.5) | 3.82 | **0.0001** |
| Glucose (mmol/L) | 6.9 (5.8–8.9) | 6.5 (5.8–9.1) | 0.43 | 0.6658 |
| A1c (%)(NGSP) | 5.8 (5.5–6.2) | 6.1 (5.6–6.6) | 2.1 | **0.0333** |
| TC (mmol/L) | 5.61 (54.95-6.20) | 5.46 (4.74-6.17) | 0.62 | 0.5353 |
| HDL (mmol/L) | 1.66 (1.37–1.97) | 1.46 (1.20–1.80) | 2.09 | **0.0371** |
| TG (mmol/L) | 1.41 (0.92–2.20) | 1.20 (0.84–2.13) | 0.64 | 0.5195 |
| LDL (mmol/L) | 3.20 (2.23–3.67) | 3.18 (2.51–3.68) | 0.37 | 0.7110 |
| DGLA (μmol/L) | 108.4 (91.9–141.6) | 106.6 (80.4–137.8) | 0.763 | 0.4453 |
| EPA% | 1.7(1.2–2.5) | 2.2 (1.3–3.3) | 1.79 | 0.0737 |
| DHA% | 4.1 (3.1–5.3) | 4.3 (3.2–5.2) | 0.37 | 0.7095 |
| History of DL drugs | 6 (12.5%) | 30 (26.8%) | 4.27 | **0.0387** |
| History of anti-HT drugs | 13 (27.1%) | 48 (42.9%) | 1.98 | 0.1595 |
| History of diabetes drugs | 3 (6.3%) | 16 (14.3%) | 2.31 | 0.1288 |
| NIHSS at admission | 8 (3–19) | 2 (1–4) | 4.79 | **< 0.0001** |
| NIHSS at discharge | 5 (2–12) | 1 (0–3) | 4.33 | **< 0.0001** |

All values except for categorical data are represented as median (interquartile range). Boldface indicates statistical significance (*p*<0.05). A1c, glycated hemoglobin; AIS, acute ischemic stroke; BMI, body mass index; chi, chi-square value; DL, dyslipidemia; EPA, eicosapentaenoic acid; DHA, docosahexaenoic acid; DBP, diastolic blood pressure; DGLA, dihomo-gamma-linolenic acid; HDL, high-density lipoprotein cholesterol; HT, hypertensive; ICH, intracerebral hemorrhage; LDL, low-density lipoprotein cholesterol; NGSP, National Glycohemoglobin Standardization Program; n, number; NIHSS, National Institutes of Health Stroke Scale score; *p*, probability; SBP, systolic blood pressure; TC, total cholesterol; TG, triglycerides; |z|, absolute value of the Wilcoxon rank-sum test statistic.
